# Crystal structures of methyl 3,5-di­bromo-4-cyano­benzoate and methyl 3,5-di­bromo-4-iso­cyano­benzoate

**DOI:** 10.1107/S2056989018002256

**Published:** 2018-02-13

**Authors:** Wayland E. Noland, Ryan J. Herzig, Abigail J. Engwall, Renee C. Jensen, Kenneth J. Tritch

**Affiliations:** a Department of Chemistry, University of Minnesota, 207 Pleasant St SE, Minneapolis, MN 55455, USA

**Keywords:** crystal structure, nitrile, isocyanide, C=O⋯Br contacts, C≡N⋯Br contacts, N≡C⋯C contacts

## Abstract

The title crystals, C_9_H_5_Br_2_NO_2_, are the first reported 2,6-dihalophenyl cyanide–isocyanide pair that have neither three- nor two-dimensional isomorphism. Both crystals contain contacts between the carbonyl O atom and a Br atom. In the crystal of the cyanide, *R*_2_^2^(10) inversion dimers form based on C≡N⋯Br contacts, a common packing feature in this series of crystals. In the isocyanide, the corresponding N≡C⋯Br contacts are not observed. Instead, the iso­cyano C atom forms contacts with the meth­oxy C atom. RNC was refined as a two-component pseudo-merohedral twin.

## Abstract 2

Even though they contain isosteric and isoelectronic mol­ecules that both form C=O⋯Br contacts, the cyanide and isocyanide crystals are not isomorphous in any dimension.

## Chemical context & database survey   

**Figure 1:**
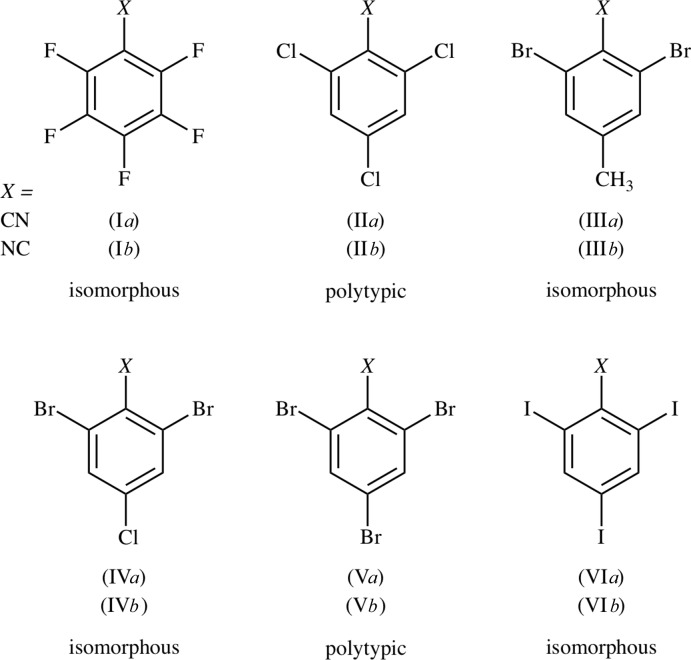
The six pairs of 2,6-dihalophenyl cyanides (_*a*) and isocyanides (_*b*) previously reported in the CSD. All corresponding crystal pairs are either isomorphous or polytypic.

The crystal packing of 2,6-dihalophenyl cyanides and isocyanides is commonly influenced by C≡N⋯*X* or N≡C⋯*X* contacts, especially when *X* is Br or I (Desiraju & Harlow, 1989 ▸). The crystal structures of isomeric, non-ligand cyanides and isocyanides are usually very similar. There are six reported 2,6-dihalophenyl cyanide–isocyanide pairs (Fig. 1 ▸). Three are in the most recent update of the Cambridge Structural Database (CSD, Version 5.38, May 2017; Groom *et al.*, 2016 ▸), and three were recently completed by our group. The penta­fluoro [(I*a*); Bond *et al.*, 2001 ▸) and (I*b*); Lentz & Preugschat, 1993 ▸)], 2,6-di­bromo-4-methyl [(III*a*), (III*b*); Noland *et al.*, 2017*b* ▸], 2,6-di­bromo-4-chloro [(IV*a*); Britton, 2005 ▸ and (IIV*b*); Noland & Tritch, 2018 ▸], and 2,4,6-tri­iodo [(VI*a*), (VI*b*); Noland *et al.* 2018 ▸] pairs are each isomorphous. The 2,4,6-tri­chloro [(II*a*), (II*b*); Pink *et al.*, 2000 ▸] and 2,4,6-tri­bromo [(V*a*), (V*b*); Britton *et al.*, 2016 ▸] pairs each have two-dimensional isomorphism and are polytypic.
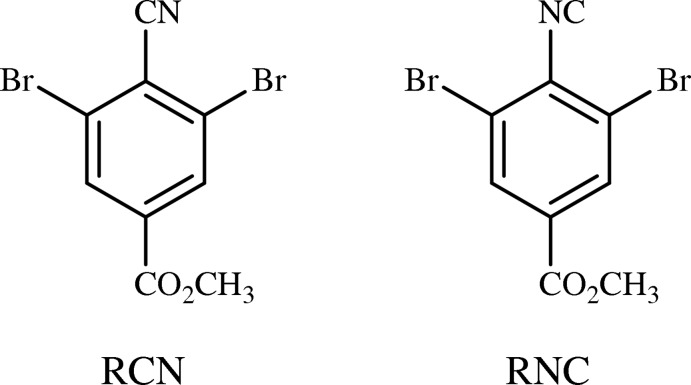


**Figure 2:**
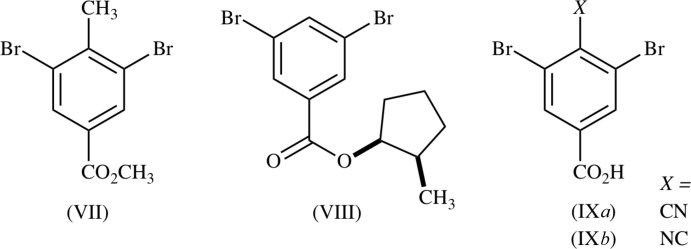
3,5-Di­bromo­benzoates (VII) and (VIII) in the CSD. We recently reported (IX*a*); iso­cyano acid (IX*b*) was not observed.

Two simple 3,5-di­bromo­benzoate esters were found in the CSD (Fig. 2 ▸). Crystals of (VII) contain *C*(6) chains of C=O⋯Br contacts (Saeed *et al.*, 2010 ▸), and crystals of (VIII) contain *C*(5) chains of Br⋯Br contacts (Reinhold & Rosati, 1994 ▸). A co-crystal of cyano acid (IX*a*) with anthracene was recently reported by our group (Noland *et al.* 2017*a* ▸). The corresponding iso­cyano acid (IX*b*) was not observed, probably because of the acid sensitivity of isocyanides (Ugi *et al.*, 1965 ▸), preventing crystallographic comparison of (IX*a*) and (IX*b*). The title cyanide (RCN) and isocyanide (RNC) were synthetic inter­mediates to (IX*a*) and (IX*b*), and their crystals are presented instead.

## Structural commentary   

**Figure 3:**
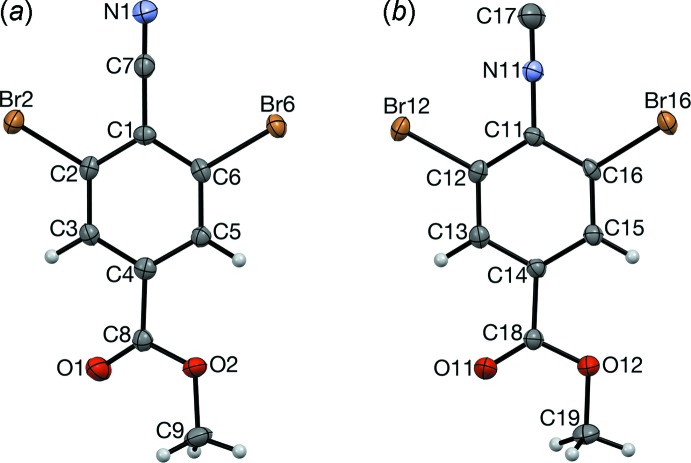
The mol­ecular structures of (*a*) RCN and (*b*) RNC, with atom labeling and displacement ellipsoids at the 50% probability level.

Mol­ecules of RCN and RNC (Fig. 3 ▸) occupy general positions and have similar, typical geometry. Both benzene rings are nearly planar, with mean atomic deviations of 0.005 (2) and 0.002 (3) Å for RCN and RNC, respectively. The most prominent difference between the mol­ecular conformations is the bond angles about the meth­oxy O atoms, which are 117.1 (2)° for C8—O2—C9, and 114.8 (3)° for C18—O12—C19. In RNC, the compression about O12 is probably caused by repulsion between methyl groups in adjacent mol­ecules, rather than the N11≡C17⋯C19 short contact (Table 1 ▸), because the C9—O2 and C19—O12 bond lengths are nearly identical.

## Supra­molecular features   

**Figure 4:**
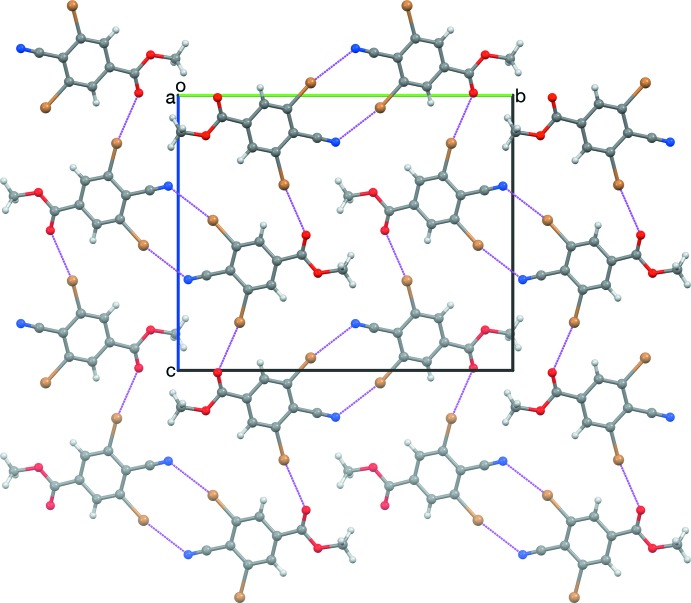
The sheet structure in a crystal of RCN, viewed along [100]. Dashed magenta lines represent short contacts.

**Figure 5:**
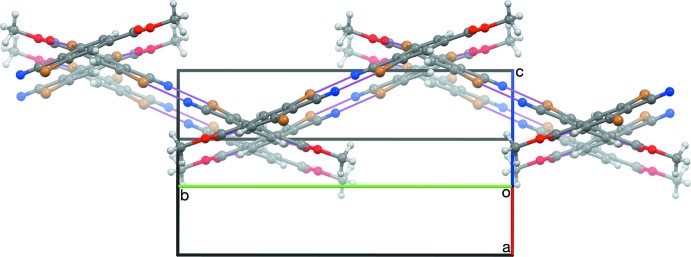
The sheet structure in a crystal of RCN, viewed along [503]. The same mol­ecules are shown as in Fig. 4 ▸.

**Figure 6:**
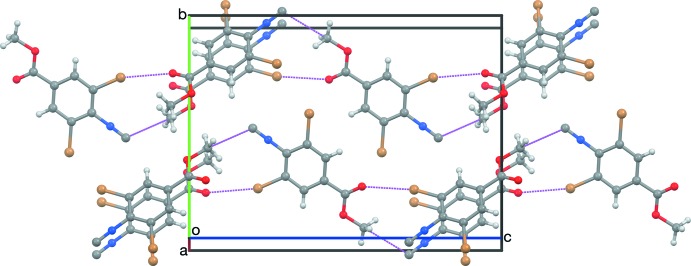
A slice of a crystal of RNC parallel to (100), viewed nearly along [100].

Mol­ecules of RCN form 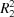
(10) inversion dimers based on C1≡N1⋯Br2 short contacts (Table 1 ▸), similar to the centric contacts found in crystals of (II) and (IV)–(VI). Adjacent dimers are connected along [201] by C8=O1⋯Br6 contacts similar to those found in (VII). Adjacent dimers are mutually inclined by 44.03 (7)°. The resulting sheet structure (Fig. 4 ▸) is staggered so that the methyl groups are spread apart to minimize steric congestion (Fig. 5 ▸). Crystals of RNC have a different packing motif, a slice of which is anti­parallel ribbons parallel to [001] (Fig. 6 ▸). Each mol­ecule of RNC participates in four short contacts between two pairs of mol­ecules that are related by the (*x* + 1, *y*, *z*) translation, forming a three-dimensional network. Contacted mol­ecules are mutually inclined by 42.0 (1)°. Half of the contacts are C18=O11⋯Br16 contacts, similar to those found in RCN and (VII). The other half are N11≡C17⋯C19 contacts, instead of the anti­cipated N11≡C17⋯Br12 contacts. It is inter­esting that the cyano group in RCN favors contacting a Br atom, but the iso­cyano group in RNC favors contacting the meth­oxy C atom.

## Synthesis and crystallization   

**Figure 7:**
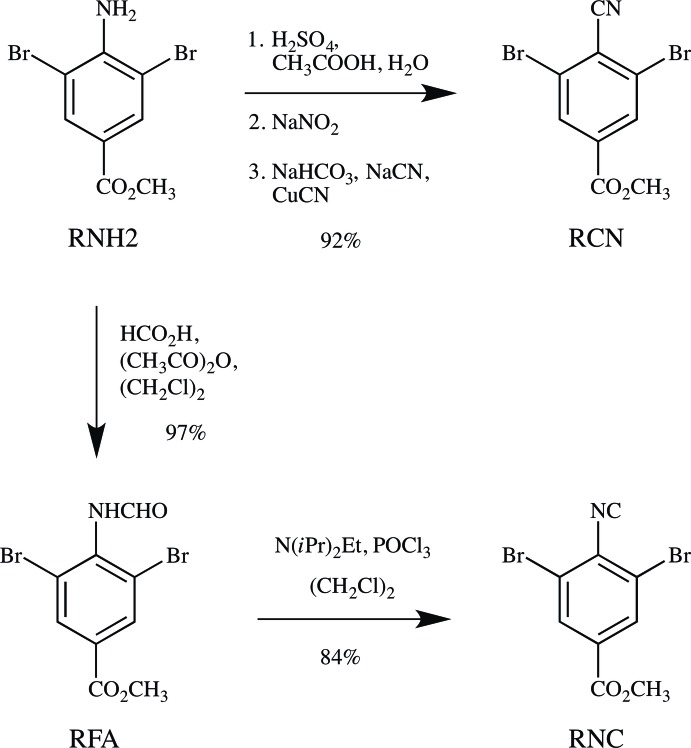
The synthesis of RCN and RNC.

**Methyl 4-amino-3,5-di­bromo­benzoate (RNH2)** and **methyl 3,5-di­bromo-4-cyano­benzoate (RCN)** were taken from material prepared in our recent study (Noland *et al.* 2017*a* ▸; Fig. 7 ▸).

**Methyl 3,5-di­bromo-4-formamido­benzoate (RFA)** was prepared from (RNH2, 1.24 g) by the formyl­ation procedure described by Britton *et al.* (2016 ▸), with 1,2-di­chloro­ethane in place of tetra­hydro­furan, giving white needles (1.31 g, 97%). M.p. 489–490 K; ^1^H NMR (300 MHz, (CD_3_)_2_CO) *δ* 9.203 (*s*, 1H), 8.441 (*s*, 1H), 8.226 (*s*, 2H), 3.928 (*s*, 3H); ^13^C NMR (126 MHz, (CD_3_)_2_SO) *δ* 163.5 (1C), 160.2 (1C), 139.5 (1C), 132.5 (2C), 130.7 (1C), 123.5 (2C), 52.9 (1C); IR (KBr, cm^−1^) 3153, 1727, 1664, 1524, 1282, 1154, 966, 765, 749; MS–ESI [*M* + Na]^+^ calculated for C_9_H_7_^79^Br^81^BrNO_3_ 359.8664, found 359.8662.

**Methyl 3,5-di­bromo-4-iso­cyano­benzoate (RNC)** was prepared from (RFA, 594 mg) by the dehydration procedure described by Britton *et al.* (2016 ▸), giving a brown powder (490 mg), which was crystallized as described below (453 mg, 84%). M.p. 391–392 K; ^1^H NMR (500 MHz, CD_2_Cl_2_) *δ* 8.278 (*s*, H13*A*, H15*A*), 3.930 (*s*, H19*A*, H19*B*, H19*C*); ^13^C NMR (126 MHz, (CD_3_)_2_SO) *δ* 174.1 (C17), 163.0 (C18), 132.5 (C13, C15), 132.3 (C14), 130.1 (C11), 121.0 (C12, C16), 53.2 (C19); IR (KBr, cm^−1^) 3073, 2961, 2853, 2122, 1722, 1426, 1275, 971, 764, 753; MS–EI [*M*]^+^ calculated for C_9_H_5_^79^Br^81^BrNO_2_ 316.8682, found 316.8699.

**Crystallization:** Crystals of RCN and RNC were grown by slow evaporation of solutions in di­chloro­methane–pentane, followed by deca­ntation, washing with pentane, and then drying at room temperature and reduced pressure (10 Pa, 4 h). RCN was obtained as colorless blocks, and RNC was obtained as colorless needles.

## Refinement   

Crystal data, data collection and structure refinement details are summarized in Table 2 ▸. A direct-methods solution was calculated, followed by full-matrix least squares/difference-Fourier cycles. All H atoms were placed in calculated positions and refined as riding atoms. For aryl H atoms, C—H = 0.95 Å and *U*_iso_(H) = 1.2*U*_eq_(C). For methyl H atoms, C—H = 0.98 Å and *U*_iso_(H) = 1.5*U*_eq_(C). RNC was refined as a two-component pseudo-merohedral twin in an 0.67:0.33 ratio, with a 180° rotation of [001] as the twinning symmetry element.

## Supplementary Material

Crystal structure: contains datablock(s) RCN, RNC. DOI: 10.1107/S2056989018002256/lh5870sup1.cif

Structure factors: contains datablock(s) RCN. DOI: 10.1107/S2056989018002256/lh5870RCNsup2.hkl

Structure factors: contains datablock(s) RNC. DOI: 10.1107/S2056989018002256/lh5870RNCsup3.hkl

Supporting information file. DOI: 10.1107/S2056989018002256/lh5870RCNsup4.cml

Supporting information file. DOI: 10.1107/S2056989018002256/lh5870RNCsup5.cml

CCDC references: 1525814, 1525813

Additional supporting information:  crystallographic information; 3D view; checkCIF report

## Figures and Tables

**Table 1:** Contact geometry for RCN and RNC (Å, °)

*A—B⋯C*	*A—B*	*B⋯C*	*A—B⋯C*
C1≡N1⋯Br2^i^	1.138 (3)	3.041 (3)	128.6 (2)
C8=O1⋯Br6^ii^	1.201 (3)	3.025 (2)	143.7 (2)
N11≡C17⋯C19^iii^	1.162 (5)	3.240 (6)	112.9 (3)
C18=O11⋯Br16^iv^	1.207 (5)	3.133 (3)	146.6 (3)

Symmetry codes: (i) −*x* + 1, −*y* + 1, −*z* + 2; (ii) *x* + 1, −*y* + 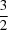, *z* + 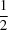; (iii) *x* + 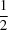, −*y* + 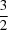, *z* + 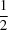; (iv) *x* + 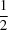, −*y* + 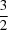, *z* − 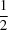.

**Table 2:** Experimental details

	RCN	RNC
Crystal data		
Chemical formula	C_9_H_5_Br_2_NO_2_	C_9_H_5_Br_2_NO_2_
*M*_r_	318.96	318.96
Crystal system, space group	Monoclinic, *P*2_1_/*c*	Monoclinic, *P*2_1_/*n*
Temperature (K)	173	173
*a*, *b*, *c* (Å)	3.9273 (18), 17.881 (8), 14.739 (7)	3.9233 (9), 13.554 (3), 18.672 (4)
β (°)	93.757 (7)	90.002 (3)
*V* (Å^3^)	1032.9 (8)	992.9 (4)
*Z*	4	4
Radiation type	Mo *K*α	Mo *K*α
μ (mm^−1^)	7.82	8.13
Crystal size (mm)	0.32 × 0.27 × 0.25	0.50 × 0.12 × 0.03

Data collection		
Diffractometer	Bruker APEXII CCD	Bruker APEXII CCD
Absorption correction	Multi-scan (*SADABS*; Sheldrick, 1996 ▸)	Multi-scan (*SADABS*; Sheldrick, 1996 ▸)
*T*_min_, *T*_max_	0.414, 0.746	0.418, 0.746
No. of measured, independent and observed [*I* > 2σ(*I*)] reflections	11889, 2426, 2013	11400, 2277, 2132
*R*_int_	0.043	0.053
(sin θ/λ)_max_ (Å^−1^)	0.657	0.650

Refinement		
*R*[*F* ^2^ > 2σ(*F* ^2^)], *wR*(*F* ^2^), *S*	0.026, 0.059, 1.07	0.029, 0.069, 1.02
No. of reflections	2426	2277
No. of parameters	128	129
H-atom treatment	H-atom parameters constrained	H-atom parameters constrained
Δρ_max_, Δρ_min_ (e Å^−3^)	0.37, −0.52	0.85, −0.65

Computer programs: *APEX2* and *SAINT* (Bruker, 2012 ▸), *SHELXT2014* (Sheldrick 2015*a* ▸), *SHELXL2014* (Sheldrick, 2015*b* ▸), *Mercury* (Macrae *et al.*, 2008 ▸) and *publCIF* (Westrip, 2010 ▸).

## supplementary crystallographic information

### Methyl 3,5-dibromo-4-cyanobenzoate (RCN) Crystal data

**Table:**

C_9_H_5_Br_2_NO_2_	*D*_x_ = 2.051 Mg m^−^^3^
*M**_r_* = 318.96	Melting point: 410 K
Monoclinic, *P*2_1_/*c*	Mo *K*α radiation, λ = 0.71073 Å
*a* = 3.9273 (18) Å	Cell parameters from 2977 reflections
*b* = 17.881 (8) Å	θ = 2.7–27.6°
*c* = 14.739 (7) Å	µ = 7.82 mm^−^^1^
β = 93.757 (7)°	*T* = 173 K
*V* = 1032.9 (8) Å^3^	Block, colourless
*Z* = 4	0.32 × 0.27 × 0.25 mm
*F*(000) = 608	

### Methyl 3,5-dibromo-4-cyanobenzoate (RCN) Data collection

**Table:**

Bruker APEXII CCD diffractometer	2013 reflections with *I* > 2σ(*I*)
Radiation source: sealed tube	*R*_int_ = 0.043
φ and ω scans	θ_max_ = 27.8°, θ_min_ = 1.8°
Absorption correction: multi-scan (SADABS; Sheldrick, 1996)	*h* = −5→5
*T*_min_ = 0.414, *T*_max_ = 0.746	*k* = −23→23
11889 measured reflections	*l* = −19→19
2426 independent reflections	

### Methyl 3,5-dibromo-4-cyanobenzoate (RCN) Refinement

**Table:**

Refinement on *F*^2^	0 restraints
Least-squares matrix: full	Hydrogen site location: inferred from neighbouring sites
*R*[*F*^2^ > 2σ(*F*^2^)] = 0.026	H-atom parameters constrained
*wR*(*F*^2^) = 0.059	*w* = 1/[σ^2^(*F*_o_^2^) + (0.0274*P*)^2^ + 0.0295*P*] where *P* = (*F*_o_^2^ + 2*F*_c_^2^)/3
*S* = 1.07	(Δ/σ)_max_ = 0.001
2426 reflections	Δρ_max_ = 0.37 e Å^−^^3^
128 parameters	Δρ_min_ = −0.52 e Å^−^^3^

### Methyl 3,5-dibromo-4-cyanobenzoate (RCN) Special details

**Table:**

Experimental. Dr. K.J. Tritch / Prof. W.E. Noland
Geometry. All esds (except the esd in the dihedral angle between two l.s. planes) are estimated using the full covariance matrix. The cell esds are taken into account individually in the estimation of esds in distances, angles and torsion angles; correlations between esds in cell parameters are only used when they are defined by crystal symmetry. An approximate (isotropic) treatment of cell esds is used for estimating esds involving l.s. planes.

### Methyl 3,5-dibromo-4-cyanobenzoate (RCN) Fractional atomic coordinates and isotropic or equivalent isotropic displacement parameters (Å^2^)

**Table:**

	*x*	*y*	*z*	*U*_iso_*/*U*_eq_	
C1	0.6370 (6)	0.65590 (14)	0.86321 (17)	0.0196 (5)	
C2	0.7731 (6)	0.67398 (14)	0.95078 (16)	0.0199 (5)	
Br2	0.74408 (8)	0.60384 (2)	1.04534 (2)	0.02954 (9)	
C3	0.9245 (6)	0.74304 (14)	0.96807 (17)	0.0204 (5)	
H3A	1.0169	0.7548	1.0275	0.024*	
C4	0.9412 (6)	0.79516 (14)	0.89815 (17)	0.0197 (5)	
C5	0.8100 (6)	0.77825 (14)	0.81049 (16)	0.0193 (5)	
H5A	0.8216	0.8138	0.7629	0.023*	
C6	0.6624 (6)	0.70889 (15)	0.79351 (16)	0.0193 (5)	
Br6	0.49149 (7)	0.68383 (2)	0.67451 (2)	0.02395 (9)	
C7	0.4695 (7)	0.58481 (16)	0.84529 (18)	0.0251 (6)	
N1	0.3333 (6)	0.52966 (14)	0.82991 (16)	0.0359 (6)	
C8	1.1066 (6)	0.86917 (14)	0.92064 (17)	0.0203 (5)	
O1	1.2632 (5)	0.88220 (11)	0.99159 (14)	0.0357 (5)	
O2	1.0590 (5)	0.91760 (10)	0.85308 (13)	0.0292 (5)	
C9	1.2078 (8)	0.99150 (15)	0.8667 (2)	0.0326 (7)	
H9A	1.1628	1.0215	0.8115	0.049*	
H9B	1.4548	0.9868	0.8798	0.049*	
H9C	1.1063	1.0161	0.9179	0.049*	

### Methyl 3,5-dibromo-4-cyanobenzoate (RCN) Atomic displacement parameters (Å^2^)

**Table:**

	*U*^11^	*U*^22^	*U*^33^	*U*^12^	*U*^13^	*U*^23^
C1	0.0183 (13)	0.0179 (13)	0.0226 (13)	0.0008 (11)	0.0018 (10)	−0.0010 (11)
C2	0.0187 (13)	0.0237 (14)	0.0174 (12)	0.0018 (11)	0.0022 (10)	0.0039 (10)
Br2	0.03760 (18)	0.02881 (17)	0.02189 (15)	−0.00681 (12)	−0.00044 (12)	0.00724 (11)
C3	0.0225 (13)	0.0220 (14)	0.0164 (12)	0.0024 (11)	−0.0001 (10)	−0.0013 (10)
C4	0.0174 (13)	0.0198 (13)	0.0219 (13)	0.0036 (10)	0.0021 (10)	−0.0002 (10)
C5	0.0213 (14)	0.0189 (14)	0.0177 (13)	0.0009 (10)	0.0011 (10)	0.0011 (10)
C6	0.0164 (13)	0.0256 (14)	0.0158 (12)	0.0028 (10)	0.0002 (10)	−0.0029 (10)
Br6	0.02657 (15)	0.02717 (16)	0.01735 (14)	−0.00102 (11)	−0.00436 (10)	−0.00197 (10)
C7	0.0273 (15)	0.0289 (16)	0.0189 (13)	−0.0006 (12)	0.0009 (11)	0.0023 (11)
N1	0.0486 (17)	0.0303 (15)	0.0282 (13)	−0.0132 (12)	−0.0021 (11)	0.0024 (11)
C8	0.0214 (13)	0.0207 (14)	0.0187 (13)	0.0020 (11)	0.0014 (10)	0.0007 (10)
O1	0.0487 (13)	0.0283 (11)	0.0283 (11)	−0.0060 (10)	−0.0112 (10)	0.0008 (9)
O2	0.0403 (12)	0.0195 (10)	0.0270 (10)	−0.0074 (9)	−0.0036 (9)	0.0025 (8)
C9	0.0387 (17)	0.0196 (15)	0.0388 (17)	−0.0077 (12)	−0.0018 (13)	0.0053 (12)

### Methyl 3,5-dibromo-4-cyanobenzoate (RCN) Geometric parameters (Å, º)

**Table:**

C1—C2	1.402 (4)	C5—H5A	0.9500
C1—C6	1.406 (4)	C6—Br6	1.890 (2)
C1—C7	1.448 (4)	C7—N1	1.138 (3)
C2—C3	1.387 (3)	C8—O1	1.201 (3)
C2—Br2	1.884 (3)	C8—O2	1.324 (3)
C3—C4	1.394 (3)	O2—C9	1.454 (3)
C3—H3A	0.9500	C9—H9A	0.9800
C4—C5	1.393 (3)	C9—H9B	0.9800
C4—C8	1.502 (4)	C9—H9C	0.9800
C5—C6	1.385 (4)		
			
C2—C1—C6	118.4 (2)	C5—C6—C1	121.4 (2)
C2—C1—C7	120.8 (2)	C5—C6—Br6	119.93 (19)
C6—C1—C7	120.8 (2)	C1—C6—Br6	118.71 (19)
C3—C2—C1	120.5 (2)	N1—C7—C1	178.6 (3)
C3—C2—Br2	120.19 (19)	O1—C8—O2	124.6 (2)
C1—C2—Br2	119.32 (19)	O1—C8—C4	123.6 (2)
C2—C3—C4	120.0 (2)	O2—C8—C4	111.9 (2)
C2—C3—H3A	120.0	C8—O2—C9	117.1 (2)
C4—C3—H3A	120.0	O2—C9—H9A	109.5
C5—C4—C3	120.5 (2)	O2—C9—H9B	109.5
C5—C4—C8	121.6 (2)	H9A—C9—H9B	109.5
C3—C4—C8	117.8 (2)	O2—C9—H9C	109.5
C6—C5—C4	119.2 (2)	H9A—C9—H9C	109.5
C6—C5—H5A	120.4	H9B—C9—H9C	109.5
C4—C5—H5A	120.4		
			
C6—C1—C2—C3	0.9 (4)	C4—C5—C6—Br6	−178.62 (18)
C7—C1—C2—C3	−178.1 (2)	C2—C1—C6—C5	−1.6 (4)
C6—C1—C2—Br2	−179.76 (18)	C7—C1—C6—C5	177.4 (2)
C7—C1—C2—Br2	1.2 (3)	C2—C1—C6—Br6	178.18 (18)
C1—C2—C3—C4	0.2 (4)	C7—C1—C6—Br6	−2.8 (3)
Br2—C2—C3—C4	−179.11 (18)	C5—C4—C8—O1	−169.5 (3)
C2—C3—C4—C5	−0.7 (4)	C3—C4—C8—O1	10.1 (4)
C2—C3—C4—C8	179.8 (2)	C5—C4—C8—O2	10.4 (3)
C3—C4—C5—C6	0.0 (4)	C3—C4—C8—O2	−170.0 (2)
C8—C4—C5—C6	179.5 (2)	O1—C8—O2—C9	0.0 (4)
C4—C5—C6—C1	1.2 (4)	C4—C8—O2—C9	−179.9 (2)

### Methyl 3,5-dibromo-4-cyanobenzoate (RCN) Hydrogen-bond geometry (Å, º)

**Table:**

*D*—H···*A*	*D*—H	H···*A*	*D*···*A*	*D*—H···*A*
C3—H3*A*···Br6^i^	0.95	2.97	3.878 (3)	160

Symmetry code: (i) *x*+1, −*y*+3/2, *z*+1/2.

### Methyl 3,5-dibromo-4-isocyanobenzoate (RNC) Crystal data

**Table:**

C_9_H_5_Br_2_NO_2_	*D*_x_ = 2.134 Mg m^−^^3^
*M**_r_* = 318.96	Melting point: 391 K
Monoclinic, *P*2_1_/*n*	Mo *K*α radiation, λ = 0.71073 Å
*a* = 3.9233 (9) Å	Cell parameters from 2953 reflections
*b* = 13.554 (3) Å	θ = 2.2–27.4°
*c* = 18.672 (4) Å	µ = 8.13 mm^−^^1^
β = 90.002 (3)°	*T* = 173 K
*V* = 992.9 (4) Å^3^	Needle, colourless
*Z* = 4	0.50 × 0.12 × 0.03 mm
*F*(000) = 608	

### Methyl 3,5-dibromo-4-isocyanobenzoate (RNC) Data collection

**Table:**

Bruker APEXII CCD diffractometer	2132 reflections with *I* > 2σ(*I*)
Radiation source: sealed tube	*R*_int_ = 0.053
φ and ω scans	θ_max_ = 27.5°, θ_min_ = 1.1°
Absorption correction: multi-scan (SADABS; Sheldrick, 1996)	*h* = −5→5
*T*_min_ = 0.418, *T*_max_ = 0.746	*k* = −17→17
11400 measured reflections	*l* = −24→24
2277 independent reflections	

### Methyl 3,5-dibromo-4-isocyanobenzoate (RNC) Refinement

**Table:**

Refinement on *F*^2^	0 restraints
Least-squares matrix: full	Hydrogen site location: inferred from neighbouring sites
*R*[*F*^2^ > 2σ(*F*^2^)] = 0.029	H-atom parameters constrained
*wR*(*F*^2^) = 0.069	*w* = 1/[σ^2^(*F*_o_^2^) + (0.0385*P*)^2^] where *P* = (*F*_o_^2^ + 2*F*_c_^2^)/3
*S* = 1.02	(Δ/σ)_max_ = 0.001
2277 reflections	Δρ_max_ = 0.85 e Å^−^^3^
129 parameters	Δρ_min_ = −0.65 e Å^−^^3^

### Methyl 3,5-dibromo-4-isocyanobenzoate (RNC) Special details

**Table:**

Experimental. Dr. K.J. Tritch / Prof. W.E. Noland
Geometry. All esds (except the esd in the dihedral angle between two l.s. planes) are estimated using the full covariance matrix. The cell esds are taken into account individually in the estimation of esds in distances, angles and torsion angles; correlations between esds in cell parameters are only used when they are defined by crystal symmetry. An approximate (isotropic) treatment of cell esds is used for estimating esds involving l.s. planes.
Refinement. Refined as a 2-component pseudo-merohedral twin in an 0.67:0.33 ratio.

### Methyl 3,5-dibromo-4-isocyanobenzoate (RNC) Fractional atomic coordinates and isotropic or equivalent isotropic displacement parameters (Å^2^)

**Table:**

	*x*	*y*	*z*	*U*_iso_*/*U*_eq_	
C11	0.5312 (10)	0.5930 (3)	0.6880 (2)	0.0187 (7)	
C12	0.6653 (9)	0.5605 (2)	0.6228 (2)	0.0193 (7)	
Br12	0.85319 (11)	0.43306 (2)	0.61597 (2)	0.02497 (11)	
C13	0.6606 (10)	0.6211 (2)	0.5634 (2)	0.0216 (8)	
H13A	0.7504	0.5983	0.5192	0.026*	
C14	0.5246 (10)	0.7153 (2)	0.56835 (19)	0.0188 (8)	
C15	0.3900 (9)	0.7501 (2)	0.63284 (19)	0.0185 (8)	
H15A	0.2971	0.8147	0.6360	0.022*	
C16	0.3944 (9)	0.6887 (3)	0.69219 (18)	0.0181 (7)	
Br16	0.21859 (11)	0.73312 (2)	0.78028 (2)	0.02528 (11)	
N11	0.5307 (9)	0.5321 (2)	0.74742 (17)	0.0240 (7)	
C17	0.5282 (15)	0.4800 (3)	0.7968 (2)	0.0402 (11)	
C18	0.5255 (11)	0.7760 (2)	0.5011 (2)	0.0210 (8)	
O11	0.6712 (9)	0.75012 (19)	0.44732 (15)	0.0321 (7)	
O12	0.3467 (8)	0.85902 (17)	0.50681 (14)	0.0270 (6)	
C19	0.3135 (13)	0.9150 (3)	0.4408 (2)	0.0306 (9)	
H19A	0.1794	0.9747	0.4498	0.046*	
H19B	0.1984	0.8745	0.4046	0.046*	
H19C	0.5404	0.9335	0.4234	0.046*	

### Methyl 3,5-dibromo-4-isocyanobenzoate (RNC) Atomic displacement parameters (Å^2^)

**Table:**

	*U*^11^	*U*^22^	*U*^33^	*U*^12^	*U*^13^	*U*^23^
C11	0.0214 (19)	0.0192 (16)	0.0153 (18)	−0.0050 (15)	−0.0002 (15)	−0.0001 (14)
C12	0.0169 (19)	0.0166 (15)	0.0246 (19)	−0.0021 (13)	−0.0002 (18)	−0.0022 (13)
Br12	0.0280 (2)	0.01584 (15)	0.0310 (2)	0.00238 (13)	0.0011 (2)	−0.00255 (14)
C13	0.021 (2)	0.0209 (16)	0.0231 (19)	−0.0018 (15)	0.0031 (16)	−0.0034 (14)
C14	0.023 (2)	0.0176 (16)	0.0160 (18)	−0.0025 (14)	0.0008 (15)	−0.0007 (14)
C15	0.020 (2)	0.0185 (15)	0.0175 (19)	−0.0019 (13)	−0.0019 (15)	−0.0022 (13)
C16	0.0196 (19)	0.0210 (16)	0.0138 (16)	−0.0049 (14)	0.0001 (14)	−0.0060 (13)
Br16	0.0296 (2)	0.02670 (18)	0.01950 (19)	−0.00182 (15)	0.00484 (19)	−0.00694 (13)
N11	0.0310 (19)	0.0198 (15)	0.0213 (17)	−0.0029 (13)	0.0011 (14)	−0.0014 (13)
C17	0.065 (3)	0.028 (2)	0.027 (2)	−0.008 (2)	−0.003 (2)	−0.0025 (19)
C18	0.027 (2)	0.0172 (17)	0.0187 (19)	−0.0031 (14)	−0.0015 (16)	−0.0021 (14)
O11	0.048 (2)	0.0235 (12)	0.0245 (15)	0.0022 (14)	0.0087 (16)	0.0019 (10)
O12	0.0391 (17)	0.0219 (12)	0.0201 (13)	0.0055 (12)	0.0001 (13)	0.0016 (10)
C19	0.039 (3)	0.0269 (18)	0.026 (2)	0.0042 (19)	−0.003 (2)	0.0071 (15)

### Methyl 3,5-dibromo-4-isocyanobenzoate (RNC) Geometric parameters (Å, º)

**Table:**

C11—N11	1.383 (5)	C15—H15A	0.9500
C11—C12	1.396 (5)	C16—Br16	1.882 (3)
C11—C16	1.407 (5)	N11—C17	1.162 (5)
C12—C13	1.379 (5)	C18—O11	1.207 (5)
C12—Br12	1.883 (3)	C18—O12	1.331 (4)
C13—C14	1.387 (5)	O12—C19	1.454 (4)
C13—H13A	0.9500	C19—H19A	0.9800
C14—C15	1.397 (5)	C19—H19B	0.9800
C14—C18	1.501 (5)	C19—H19C	0.9800
C15—C16	1.386 (5)		
			
N11—C11—C12	120.8 (3)	C15—C16—C11	120.9 (3)
N11—C11—C16	120.3 (3)	C15—C16—Br16	120.2 (3)
C12—C11—C16	118.9 (3)	C11—C16—Br16	118.9 (3)
C13—C12—C11	120.5 (3)	C17—N11—C11	179.1 (4)
C13—C12—Br12	119.8 (3)	O11—C18—O12	124.2 (4)
C11—C12—Br12	119.7 (3)	O11—C18—C14	122.6 (3)
C12—C13—C14	120.0 (3)	O12—C18—C14	113.2 (3)
C12—C13—H13A	120.0	C18—O12—C19	114.8 (3)
C14—C13—H13A	120.0	O12—C19—H19A	109.5
C13—C14—C15	120.9 (3)	O12—C19—H19B	109.5
C13—C14—C18	116.6 (3)	H19A—C19—H19B	109.5
C15—C14—C18	122.5 (3)	O12—C19—H19C	109.5
C16—C15—C14	118.8 (3)	H19A—C19—H19C	109.5
C16—C15—H15A	120.6	H19B—C19—H19C	109.5
C14—C15—H15A	120.6		
			
N11—C11—C12—C13	179.1 (3)	C14—C15—C16—Br16	179.5 (3)
C16—C11—C12—C13	−0.6 (6)	N11—C11—C16—C15	−179.3 (4)
N11—C11—C12—Br12	−0.8 (5)	C12—C11—C16—C15	0.4 (5)
C16—C11—C12—Br12	179.5 (3)	N11—C11—C16—Br16	1.1 (5)
C11—C12—C13—C14	0.6 (6)	C12—C11—C16—Br16	−179.2 (3)
Br12—C12—C13—C14	−179.5 (3)	C13—C14—C18—O11	−8.6 (6)
C12—C13—C14—C15	−0.3 (6)	C15—C14—C18—O11	172.4 (4)
C12—C13—C14—C18	−179.3 (4)	C13—C14—C18—O12	170.5 (3)
C13—C14—C15—C16	0.0 (6)	C15—C14—C18—O12	−8.5 (6)
C18—C14—C15—C16	179.0 (3)	O11—C18—O12—C19	4.8 (6)
C14—C15—C16—C11	−0.1 (6)	C14—C18—O12—C19	−174.2 (4)
